# C_0.3_N_0.7_Ti-SiC Toughed Silicon Nitride Hybrids with Non-Oxide Additives Ti_3_SiC_2_

**DOI:** 10.3390/ma13061428

**Published:** 2020-03-20

**Authors:** Heng Luo, Chen Li, Lianwen Deng, Yang Li, Peng Xiao, Haibin Zhang

**Affiliations:** 1 School of Physics and Electronics, Central South University, Changsha 410083, China; li-richard@foxmail.com (C.L.); denglw@csu.edu.cn (L.D.); 2 State Key Laboratory of Powder Metallurgy, Central South University, Changsha 410083, China; liyang_csu@126.com (Y.L.); xiaopengcsu@csu.edu.cn (P.X.); 3 Innovation Research Team for Advanced Ceramics, Institute of Nuclear Physics and Chemistry, China Academy of Engineering Physics, Mianyang 621900, China; hbzhang@caep.cn

**Keywords:** Ti_3_SiC_2_, Si_3_N_4_, mechanical properties, fracture toughness

## Abstract

In situ grown C_0.3_N_0.7_Ti and SiC, which derived from non-oxide additives Ti_3_SiC_2_, are proposed to densify silicon nitride (Si_3_N_4_) ceramics with enhanced mechanical performance via hot-press sintering. Remarkable increase of density from 79.20% to 95.48% could be achieved for Si_3_N_4_ ceramics with 5 vol.% Ti_3_SiC_2_ when sintered at 1600 °C. As expected, higher sintering temperature 1700 °C could further promote densification of Si_3_N_4_ ceramics filled with Ti_3_SiC_2_. The capillarity of decomposed Si from Ti_3_SiC_2_, and in situ reaction between nonstoichiometric TiC*_x_* and Si_3_N_4_ were believed to be responsible for densification of Si_3_N_4_ ceramics. An obvious enhancement of flexural strength and fracture toughness for Si_3_N_4_ with *x* vol.% Ti_3_SiC_2_ (*x* = 1~20) ceramics was observed. The maximum flexural strength of 795 MPa for Si_3_N_4_ composites with 5 vol.% Ti_3_SiC_2_ and maximum fracture toughness of 6.97 MPa·m^1/2^ for Si_3_N_4_ composites with 20 vol.% Ti_3_SiC_2_ are achieved via hot-press sintering at 1700 °C. Pull out of elongated Si_3_N_4_ grains, crack bridging, crack branching and crack deflection were demonstrated to dominate enhance fracture toughness of Si_3_N_4_ composites.

## 1. Introduction

Although several alternatives of structural ceramics have been proposed, silicon nitride (Si_3_N_4_)-based ceramics remain competitive due to their superior properties, involving high strength and hardness at elevated temperatures, high resistance to oxidation and chemical attack, low coefficient of tribological friction and thermal expansion, and low dielectric permittivity, etc. [1,2,3,4,5,6,7,8,9,10]. As important multifunctional materials, Si_3_N_4_ ceramics have found wide range of successful application towards gas turbine engine components [10,11,12,13,14], cutting tools [11,15], radomes [2], and even integrated circuit [16,17], optical devices [18,19], etc.

However, due to the high degree of covalent bonding, Si_3_N_4_-based ceramics are very difficult to densify through the solid-state sintering process. Therefore, effective approaches to ensure rapid consolidation and high mechanical performance of Si_3_N_4_-based ceramics are actively being explored, including gas pressure sintering (GPS) [11], hot-pressing sintering (HPS) [20,21,22,23,24,25,26], hot isostatic pressing sintering (HIP) [27], spark plasma sintering (SPS) [26,28,29], and microwave sintering [1,30], etc. However, considering the requirement of high gas pressures for gas pressure sintering and extra current devices for SPS with a significantly higher furnace costs, HPS allows the dense and complex-shaped parts with medium cost [2]. Previous considerable efforts have demonstrated that fully dense Si_3_N_4_ ceramics with superior strength could be achieved through liquid phase sintering by addition of rare-earth oxides to promote mass transport and accelerate the rate of *α* − *β* transformation, most notably the rare-earth oxides involving Y_2_O_3_ [4,31,32]. A combination of various rare-earth oxides and other metallic oxides, such as Y_2_O_3_, La_2_O_3_, Nd_2_O_3_, Sm_2_O_3_, Yb_2_O_3_, Lu_2_O_3_, Al_2_O_3_, and MgO, also are effective sintering aids to densify Si_3_N_4_ [25,30,32,33,34,35].

Nevertheless, these oxides additives crystallized to intergranular glassy phase in the cooling stage [31], which deteriorate the high-temperature performance of the ceramics such as creep and high-temperature strength due to the relative low eutectic temperature [32,36]. As a result of the early interest in hot-pressed Si_3_N_4_ ceramics as a high-temperature gas turbine material, attention was directed to high-temperature strength and creep resistance. Therefore, it is quite essential to explore novel heat-resistant sintering aids for high-performance Si_3_N_4_ ceramics from new view point.

The last two decades have been witness to the dramatic development on MAX phase cermets with the hexagonal symmetry due to their unique combination of characteristics of both ceramics and metals (M is an early transition metal, A is a group A element, X is either carbon and/or nitrogen), especially the layered ternary carbide titanium aluminum carbide (Ti_3_AlC_2_) and titanium silicon carbide (Ti_3_SiC_2_) [37,38,39]. The crystal structure of these MAX cermets can be described by alternately stacking of TiC_6_ and Al/Si atomic planes. The unique combination of excellent properties of Ti_3_AlC_2_ or Ti_3_SiC_2_, including high melting point, high hardness, high elastic modulus, good thermal and electrical conductivity, and considerable chemical stability, make them to be fascinating candidates for various application. Moreover, elemental metal powder-derived MAX materials have demonstrated to be effective reinforcement in TiB_2_ [40,41,42], Al_2_O_3_ [43,44,45] composites with enhanced mechanical properties by in situ reaction. More recently, as explicated in our previous work [46,47,48,49,50], titanium aluminum carbide (Ti_3_AlC_2_) was chosen as an effective sintering aid to effectively densify B_4_C ceramics with enhanced sintering ability and mechanical performance simultaneously. High hardness and toughness values of 28.5 GPa and 7.02 MPa·m^1/2^ respectively were achieved for B_4_C composites sintered with 20 vol.% Ti_3_AlC_2_ at 1900 °C. The mechanisms of the enhanced sinterability of high-performance ceramics in previous works [1,10,46,47,48,49,50] could be classified into two aspects: Firstly, the decomposed metals from Ti_3_SiC_2_ or Ti_3_AlC_2_ at high-temperature can form liquid phase which promote sintering effectively. Secondly, in situ reaction sintering between matrix and titanium carbon compound would also promote densification and mechanical performance. The main competitive advantage of MAX aids is considered to be formation of reaction bonding between Si_3_N_4_ matrix and aids, rather than intergranular glassy phase. Motivated by such an idea [46,47,48,49,50], these non-oxides cermets are highly expected to play a multifunctional role in densification and enhancement of mechanical properties of Si_3_N_4_ ceramics.

It is also noteworthy that Al decomposed from Ti_3_AlC_2_ and residual O originated from raw Si_3_N_4_ powders would be dissolved into Si_3_N_4_ grains during high-temperature sintering procedure, which is harmful to the purity and thermal performance of Si_3_N_4_-based ceramics. As Y. Zhou et al. illustrated [51], a tendency of decreasing fracture toughness with increasing Al dopant could be observed. Moreover, even the 0.4 wt.% concentration of Al would lead to a drastically reduce of thermal conductivity by 36.9% (from 91.9 to 58.0 W·m^−1^·K^−1^) for Si_3_N_4_ ceramics. Therefore in this work, Ti_3_SiC_2_ were introduced to densify Si_3_N_4_ ceramics in order to demonstrate that it provided any advantages over the rare-earth oxide system. Besides, the effect of Ti_3_SiC_2_ volume fraction on the microstructure, hardness, flexural strength and fracture toughness was also studied. It is believed that Ti_3_SiC_2_ or other members of MAX family would lead to new scientific and technological data providing new insight into functionalization of Si_3_N_4_ ceramics.

## 2. Experimental Procedure

### 2.1. Preparation of Samples

Commercially available *α*-Si_3_N_4_ powder (purity > 93%, *d*_50_ = 0.7 μm, Jinshenghao New Materials Co. Ltd., Anyang, China) was used as a starting material. As a novel sintering aid, Ti_3_SiC_2_ powders (*d*_50_ = 5 μm, purity > 98%) were kindly provided by Forsman Scientific Co., Ltd., Beijing, China. To investigate the effect of Ti_3_SiC_2_ content on the mechanical properties, experiments were conducted with various amounts of Ti_3_SiC_2_ powders (1 to 20 vol.%) embedded in *α*-Si_3_N_4_ powders. To ensure the homogeneity of the mixed powders, *α*-Si_3_N_4_ with *x* vol.% Ti_3_SiC_2_ powders (*x* = 1~20) were wet ball-milled for 10 h by using ethanol as ball-milling media. The substance was dried at 80 °C, and sieved with a filter with a mesh size of 63 μm, then placed in a graphite die coated with BN powder to avoid reaction between the powder and graphite die. Hot-press (HP) sintering was performed on vacuum hot press sintering furnace (ZT-63-21Y, Shanghai Chenhua Technology Co., Ltd., Shanghai, China) with ramp of 10 °C /min 1600 °C and 1700 °C for 90 min in flowing nitrogen under 30 MPa uniaxial pressure during the whole cycle. After natural cooling to room temperature inside furnace, samples were polished and ultrasonic cleaned before characterization. For comparison, *α*-Si_3_N_4_ powders with 2 wt.% Alumina (Al_2_O_3_, AR, Sinopharm Chemical Reagent Co., Ltd., Shanghai, China) and 5 wt.% yttria (Y_2_O_3_, AR, Sinopharm Chemical Reagent Co., Ltd., Shanghai, China) were hot-pressed at the same sintering condition.

### 2.2. Characterizations

Before microstructure and mechanical performance characterizations, all hot-pressed samples with diameter of 50 mm were cut into bars and cuboids with help of inside diameter slicer. To reduce surface roughness of samples and guarantee sufficient experimental precision, the abrasive SiC papers with grit size of P320, P600, P1200 and P2000 were used in chronological order during the polishing process. Final manual polishing was carried out with polishing cloth containing alumina suspension with particle size 0.3 μm. The bulk density of each sample was determined according to the Archimedes principle in distilled water. X-ray Diffraction (XRD) patterns were recorded on X’pert PRO (PANalytical B.V., Almelo, Netherlands). Phase identification and quantitative analysis were performed on MDI Jade software (version 6.0, MDI, Livermore, CA, USA) according to Rietveld method. The microstructures of polished surfaces and fracture surfaces were observed using scanning electron microscopy (SEM, Nova NanoSEM 230, FEI Company, Hillsboro, OR, USA) with an energy dispersive X-ray (EDX) analyzer. The Vickers hardness was performed on micro hardness tester (VTD 512, Beijing Weiwei Technology Co., LTD, Beijing, China) under load of 9.8 N with a dwell time of 10 s, and determined by the Vickers diamond indentation method using the following equation:

(1) HV=0.102FS=0.1022Fsin1362d2=0.1891Fd2

where *P* is the indentation load on the polished surface and *d* is the average diagonal length of the Vickers indentation. For accuracy, 11 Vickers indentations on each specimen were applied. After indentation, the microstructures were immediately observed by optical microscopy (ECLISPE LV150N, Tokyo, Japan). As a simple way of estimating toughness, indentation techniques were applied from observed corner cracks and calculated Vickers hardness using the Anstis equation:

(2) KIC=0.016(EHV)12(Pc32)

where *E* is the Young’s modulus and *c* is the half-length of cracks formed by the indentation. According to the international standard of test method for flexural strength of fine monolithic ceramics at room temperature which described in ISO 14704-2000, three-point flexural strength of specimens with size of 3 mm × 4 mm × 36 mm was performed on the mechanical testing machine (Instron 3369, INSTRON Corporation, Norwood, MA, USA) at a cross head speed of 0.5 mm/min.

## 3. Results and Discussion

**Figure 1:**
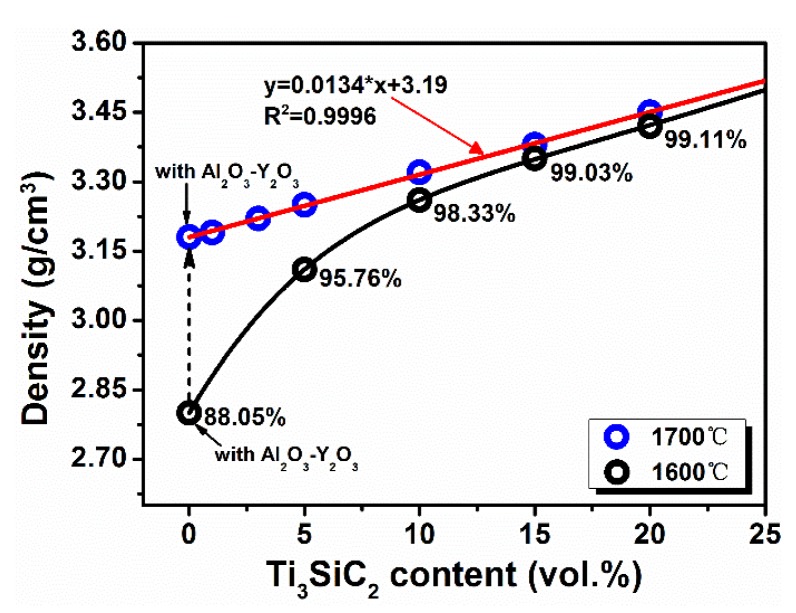
Density of Si_3_N_4_ ceramics as a function of Ti_3_SiC_2_ content.

Figure 1 illustrates the density of Ti_3_SiC_2_ filled Si_3_N_4_ ceramics as a function of Ti_3_SiC_2_ volume fraction. For Si_3_N_4_ ceramics which HP sintered at 1600 °C without aids, the density is only 2.58 g·cm^−3^. Partial densification may be attributed to the residual SiO_2_ liquid phase during firing at high-temperature which always present on Si_3_N_4_ powder particles. A remarkable increase to 3.11 g·cm^−3^ could be observed for Si_3_N_4_ ceramics filled with only 5 vol.% Ti_3_SiC_2_ when sintered at the same temperature. The enhanced density is even more noticeable than the Si_3_N_4_ ceramics with 7 wt.% Y_2_O_3_-Al_2_O_3_ aids. These observed results demonstrate Ti_3_SiC_2_ to be a effective sintering aid to densify Si_3_N_4_ ceramics. However, further increase in Ti_3_SiC_2_ content dose not bring any appreciable consolidation.

**Figure 2:**
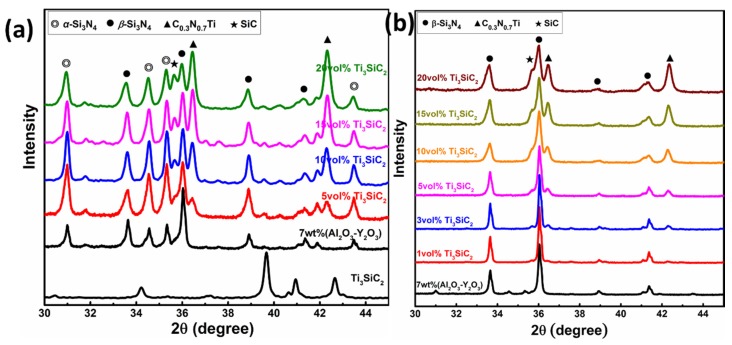
XRD patterns of Ti_3_SiC_2_ doped Si_3_N_4_ ceramics sintered at (**a**) 1600 and (**b**) 1700 °C.

As expected, higher sintering temperature 1700 °C could further promote densification of Si_3_N_4_ ceramics filled with Ti_3_SiC_2_. Besides, experimental points are inclined to distribute on a straight line with coefficient of determination (*R*^2^) above 0.99. To investigate the composition evolution after sintering, XRD patterns of Si_3_N_4_ ceramics filled with different volume fraction of Ti_3_SiC_2_ sintered at 1600 and 1700 °C, as well as 7 wt.% (Al_2_O_3_-Y_2_O_3_) densified Si_3_N_4_ ceramics are shown in Figure 2. As seen in Figure 2a, both *α* and *β* phase of Si_3_N_4_ could be detected when sintering temperature is 1600 °C, which suggests only a partial transformation of *α* phase to the more stable *β* phase. In contrast, when further improving sintering temperature to 1700 °C, all diffraction peaks of *α*-Si_3_N_4_ phase disappear (see in Figure 2b). This completely transformation of *α* to *β*-Si_3_N_4_ phase is believed to be essential to the enhancement of densification and mechanical performance [1,52].

Another important feature should be noted here is that the characteristic diffraction peaks of the raw Ti_3_SiC_2_ powder nearly disappear completely after sintering. This could be ascribed to the fact that Ti_3_SiC_2_ powder is thermal stable up to ~800 °C, and the following reaction can be responsible for the decomposition of Ti_3_SiC_2_ [53]:

(3) $$3Ti_{3}SiC_{2}\overset{\sim 800{}^{\circ}C\;to\;1400{}^{\circ}C}{\to }4TiC_{x}+Ti_{5}Si_{3}C_{y}+(6-4x-y)C$$

where the value of *x* ranges from 0.6 to 0.8 and *y* ≤ 1. Besides, the TiC*_x_* phase appears to result in more rapid deterioration of the Ti_3_SiC_2_ phase. Also noted that the decomposition usually accomplished with decomposition of Ti_3_SiC_2_ to form nonstoichiometric TiC*_x_* and gaseous Si, as demonstrated previously [54]:

(4) Ti3SiC2→~1300°C3TiC23+Si

The Si is believed to be act as lubricating phase between Si_3_N_4_ grains to promote densification of Si_3_N_4_ ceramics through capillarity. Meanwhile, nitriding of TiC*_x_* which originated from interatomic diffusion of C and N [10] during high-temperature sintering process would lead to the formation of new phase C_0.3_N_0.7_Ti. In addition, the residual Si would further react with nitrogen to form Si_3_N_4_ [55,56,57]. On the other hand, further heating during insulation stage will result in the likely loss of gaseous silicon. Therefore, it is reasonable to assume that the decomposition products of Ti_3_SiC_2_ would further react with Si_3_N_4_ through diffusion of C and N according to the following reactions:

(5) $$Ti_{5}Si_{3}C_{y}\to Ti_{5}C_{y}+3Si$$

(6) $$TiC_{x}+Si_{3}N_{4}\to (3.333x-10)C_{0.3}N_{0.7}Ti+3SiC+(5.5-1.167x)N_{2}$$

(7) $$Ti_{5}C_{y}+(4.5-y)C+Si_{3}N_{4}\to 5C_{0.3}N_{0.7}Ti+3SiC+0.25N_{2}$$

(8) $$3C+Si_{3}N_{4}\to 3SiC+2N_{2}$$

(9) C+Si→SiC

or

(10) $$\begin{array}{c} TiC_{x}+Ti_{5}Si_{3}C_{y}+(9.45-x-y)C+1.55Si_{3}N_{4}\\ \to 6C_{0.3}N_{0.7}Ti+7.65SiC+N_{2} \end{array}$$

It is reasonable to claim that the in situ reaction is responsible for the additional characteristic diffraction peaks of C_0.3_N_0.7_Ti and SiC in XRD patterns.

Furthermore, detailed refinement parameters by means of Rietveld method are summarized in Table 1. Detailed refinement parameters, including weight fraction and *R* factor, of Ti_3_SiC_2_ doped Si_3_N_4_ ceramics are illustrated in Figures S1–S6 (see in Supplementary Materials). Obviously, the weight fraction of both C_0.3_N_0.7_Ti (Fm-3m, PDF# 42-1448) and moissanite-3C SiC (F-43m, PDF# 29-1129) are inclined to increase with Ti_3_SiC_2_ content, which in turn confirms the in situ densification sintering mechanism discussed above. In addition, theoretical density could be derived with help of Rietveld method. Results have shown that nearly full densification for Ti_3_SiC_2_ doped Si_3_N_4_ ceramics sintered at 1700 °C could be achieved.

**Figure 3:**
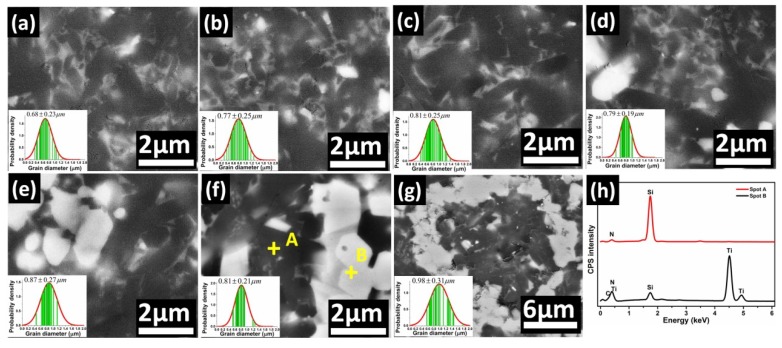
Polished surface of Si_3_N_4_ ceramics with different content of Ti_3_SiC_2_ sintered at 1700 °C: (**a**) 5 wt.% Y_2_O_3_-2 wt.% Al_2_O_3_, (**b**) 1 vol.% Ti_3_SiC_2_, (**c**) 3 vol.% Ti_3_SiC_2_, (**d**) 5 vol.% Ti_3_SiC_2_, (**e**) 10 vol.% Ti_3_SiC_2_, (**f**) 15 vol.% Ti_3_SiC_2_, (**g**) 20 vol.% Ti_3_SiC_2_, (**h**) EDS spectra at spot A and B.

Figure 3 shows the micromorphology of polished surface of Si_3_N_4_ with different volume fractions of Ti_3_SiC_2_ sintered at 1700 °C. Due to the lack of sufficient sintering aids, lots of pores could be observed, and grain growth of *β*-Si_3_N_4_ is not complete for monolithic Si_3_N_4_ ceramic (see Figure 3a). However, the microstructures of Ti_3_SiC_2_-Si_3_N_4_ ceramics (see Figure 3b–g) exhibit much more close-grain structure and consist of randomly oriented elongated Si_3_N_4_ grains which is accordant with XRD results in Figure 2. The average diameters of grains present slight increasing trend from 0.68 to 0.98 μm by quantitative image analysis as the amount of Ti_3_SiC_2_ increased. Besides, the bright contrasted phase which uniformly embedded in Si_3_N_4_ matrix could be observed and are inclined to aggregate especially when Ti_3_SiC_2_ content exceeds 15 vol.%. Furthermore, as shown in Table 2, energy dispersive spectrometer (EDS) at spot A in Figure 3e suggests dominant phase of Si_3_N_4_ and SiC, which is associated with reaction described by Equation (10). Additional O element may be originated from surface of raw *α*-Si_3_N_4_ powders. Meanwhile, the bright region at spot B is proved to be enriched by Ti according to the EDS results in Table 2. Combined with the results of XRD analysis, it is reasonable to claim that the dispersive bright regions consist of C_0.3_N_0.7_Ti and SiC, which are believed to affect the mechanical performance of reaction bonded Si_3_N_4_ ceramics.

**Figure 4:**
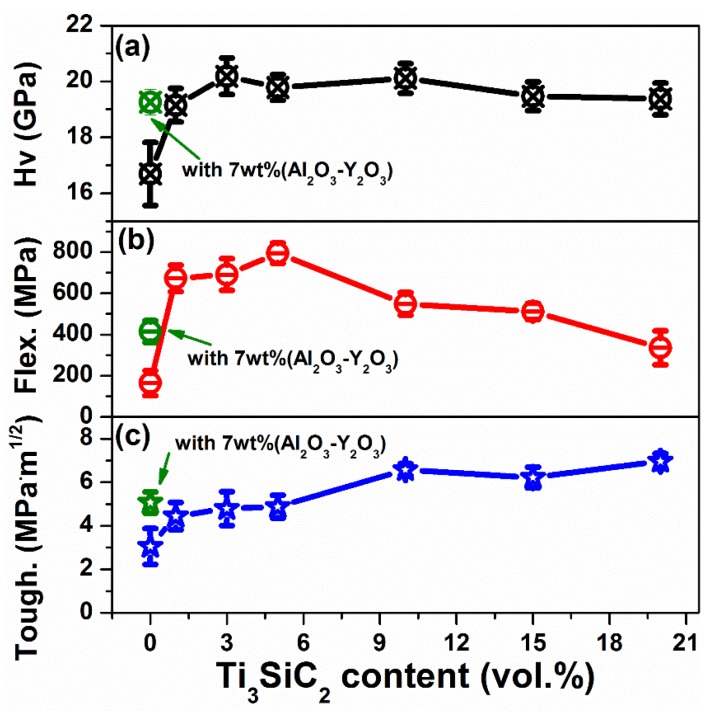
Mechanical properties of Ti_3_SiC_2_ doped Si_3_N_4_ ceramics: (**a**) Vickers hardness; (**b**) Flexural strength and (**c**) fracture toughness.

The mechanical properties, including Vickers hardness, flexural strength, and fracture toughness, of dense Si_3_N_4_ ceramics with different Ti_3_SiC_2_ content sintered at 1700 °C are illustrated in Figure 4. Clearly, the Vickers hardness of Si_3_N_4_ ceramics has been upgraded after modification of Ti_3_SiC_2_, and presents slight increase compared with that of Si_3_N_4_ ceramics containing conventional oxides aids. Besides, an obvious enhancement of flexural strength and fracture toughness could be observed. A maximum flexural strength of 795 MPa could be achieved for 5 vol.% Ti_3_SiC_2_ doped Si_3_N_4_ composites, which is almost twice that of 7 wt.% (Y_2_O_3_-Al_2_O_3_)-Si_3_N_4_ ceramics prepared at the same condition. This enhancement of flexural strength could be attributed to the C_0.3_N_0.7_Ti and SiC which originated from reaction bonding between Ti_3_SiC_2_ and Si_3_N_4_ [10]. However, further increment of Ti_3_SiC_2_ content reduces the flexural strength of Si_3_N_4_ ceramics which may be ascribed to the enhanced residual stresses around grain boundary [58,59]. Please note that this residual stress is believed to result in microcracks and intergranular fracture mode which will be discussed later. Moreover, the fracture toughness of Si_3_N_4_ composites is also effectively boosted after Ti_3_SiC_2_ decoration, and reaches maximum value of 6.97 MPa·m^1/2^ for 20 vol.% Ti_3_SiC_2_-Si_3_N_4_ ceramics which is 37% higher than that of 7 wt.% (Y_2_O_3_-Al_2_O_3_)-Si_3_N_4_ ceramics.

**Figure 5:**
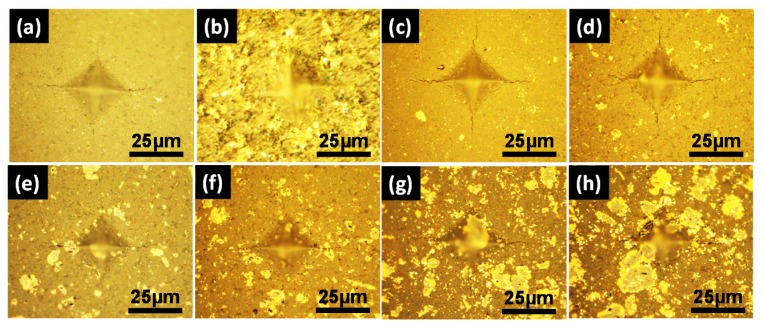
Optical micrographs of the Vickers hardness indents and the induced cracks in (**a**) Si_3_N_4_-7 wt.% (Al_2_O_3_-Y_2_O_3_), (**b**) Si_3_N_4_-3 vol.% Ti_3_SiC_2_, (**c**) Si_3_N_4_-5 vol.% Ti_3_SiC_2_, (**d**) Si_3_N_4_-10 vol.% Ti_3_SiC_2_, (**e**) Si_3_N_4_-15 vol.% Ti_3_SiC_2_, (**f**) Si_3_N_4_-20 vol.% Ti_3_SiC_2_.

Figure 5 illustrates the typical optical micrographs of the Vickers hardness indents and the induced cracks of Si_3_N_4_ ceramics with different Ti_3_SiC_2_ contents, as well as 7 wt.% (Y_2_O_3_-Al_2_O_3_). Clearly, the polished surfaces of Ti_3_SiC_2_ doped Si_3_N_4_ ceramics become much smoother than the monolithic Si_3_N_4_ ceramic which HP sintered at 1700 °C, corresponding to the enhancement of densification. Besides, it can be seen that the area of indentation presents no obvious change for Ti_3_SiC_2_ doped Si_3_N_4_ ceramics, which is consistent with the stable Vickers hardness. However, the cracks obviously become shorter especially when the Ti_3_SiC_2_ contents exceed 10 vol.%, which is responsible for the enhancement of fracture toughness.

**Figure 6:**
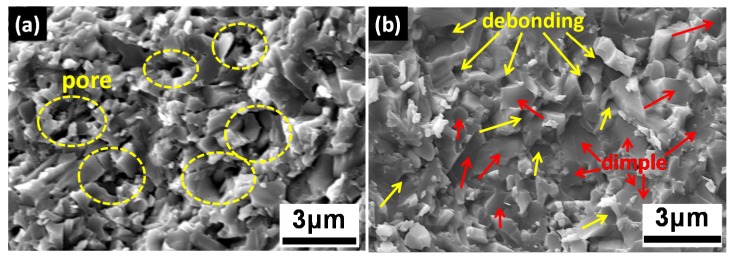
Typical fracture surfaces of Si_3_N_4_ with (**a**) 7 wt.% (Al_2_O_3_-Y_2_O_3_), (**b**) 10 vol.% Ti_3_SiC_2._

To illustrate the fracture behaviors and activated toughening mechanisms, micromorphology and crack paths are investigated on cross-sectional fracture surfaces and polished surfaces, respectively. Comparison of typical fracture surfaces between Si_3_N_4_ doped with Al_2_O_3_-Y_2_O_3_ and Ti_3_SiC_2_ is illustrated in Figure 6. As can be seen from Figure 6a, a small number of pores occur in the Si_3_N_4_-7 wt.% (Al_2_O_3_-Y_2_O_3_) composites, which is harmful for the mechanical performance. In contrast, the Si_3_N_4_-Ti_3_SiC_2_ specimen presents a much more close-grain fracture surface owning to the higher density. As marked by red arrows in Figure 6b, large amounts of dimples corresponding to the transgranular fracture could be observed. In addition, this fracture mode is considered to make a dominant contribution to the superior flexural strength of Si_3_N_4_-Ti_3_SiC_2_ composites [10]. Besides, as marked by yellow arrows, lots of interface debonding between the Si_3_N_4_ grains and grain boundary phase could be observed. This intergranular fracture mode may result from the pullout of elongated *β*-Si_3_N_4_ grains, which is believed to make a contribution to the enhancement of overall fracture toughness [60,61,62].

**Figure 7:**
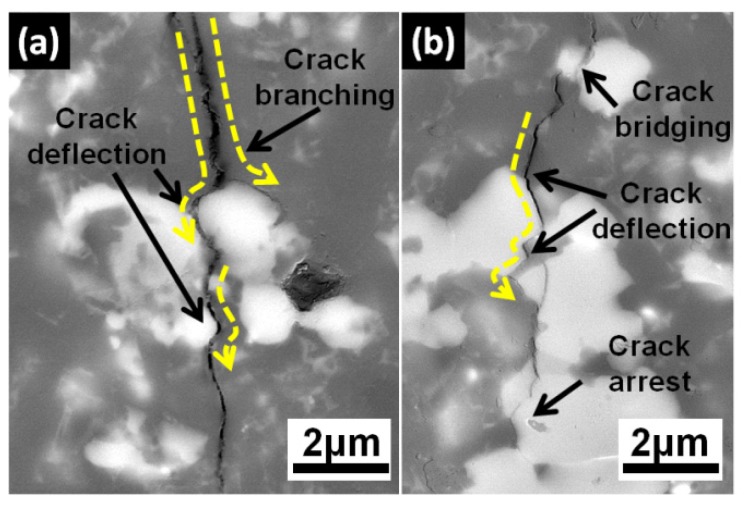
Typical SEM images of crack deflection in Ti_3_SiC_2_ densified Si_3_N_4_ composites.

Another mechanism of the enhanced fracture toughness of Si_3_N_4_-Ti_3_SiC_2_ composites could be ascribed to the crack branching, deflection, and grain bridging by in situ derived C_0.3_N_0.7_Ti and SiC grains embedded in Si_3_N_4_ matrix, which illustrated in Figure 7. Due to the superior hardness of C_0.3_N_0.7_Ti and the thermal mismatch between Si_3_N_4_ and C_0.3_N_0.7_Ti, there exists residual stress around C_0.3_N_0.7_Ti grains during cooling, giving rise to the microcracks inside composites. When subjected to the external mechanical stress, these microcracks tend to be activated and the propagation path of cracks tends to be split by C_0.3_N_0.7_Ti hard-phase and deflected along the interface. Such mechanisms consumed more fracture energy during the crack propagation which leads to crack arrest [63,64,65,66,67].

A comparison of mechanical properties of Si_3_N_4_-based ceramics obtained in the present work and selected previous works with conventional oxide aids is shown in Table 3. Clearly, the Vickers, flexural strength, and toughness of Ti_3_SiC_2_ doped Si_3_N_4_ ceramics present the same level or even better compared with Si_3_N_4_ ceramics sintered with oxide aids. Moreover, due to the superior mechanical and thermal properties of in situ formed C_0.3_N_0.7_Ti and SiC, Si_3_N_4_ ceramics obtained in this work are believed to have a significant competitive advantage and to promote the development of Si_3_N_4_-based ceramics at high temperatures.

## 4. Conclusions

In summary, we proposed non-oxide Ti_3_SiC_2_ (one of typical MAX cermets) as a novel sintering aid to densify Si_3_N_4_ ceramics with enhanced mechanical properties. A remarkable relative density increment of 20.5% (from 2.58 to 3.11 g·cm^−3^) could be observed for 1600 °C hot-press sintered Si_3_N_4_ ceramics doped with only 5 vol.% Ti_3_SiC_2_ compared with Si_3_N_4_ ceramics without aids. Further increase in sintering temperature to 1700 °C brought appreciable consolidation of nearly full dense Ti_3_SiC_2_-Si_3_N_4_ ceramics. XRD and EDS investigations demonstrated the formation of C_0.3_N_0.7_Ti and SiC which resulted from in situ reaction between Ti_3_SiC_2_ and Si_3_N_4_ through diffusion of C and N. The Vickers hardness of Ti_3_SiC_2_ doped Si_3_N_4_ ceramics increased slight compared with that of Si_3_N_4_ ceramics containing conventional oxides aids. Nevertheless, an obvious enhancement of flexural strength and fracture toughness could be observed. A maximum flexural strength of 795 MPa could be obtained for 5 vol.% Ti_3_SiC_2_ doped Si_3_N_4_ composites. Moreover, the fracture toughness of Ti_3_SiC_2_ densified Si_3_N_4_ composites exhibited a remarkable increase with increasing in volume fraction, and reached maximum value of 6.97 MPa·m^1/2^ for 20 vol.% Ti_3_SiC_2_-Si_3_N_4_ ceramics. Pull out of elongated Si_3_N_4_ grains, crack bridging and deflection were demonstrated to promote fracture toughness of Ti_3_SiC_2_ densified Si_3_N_4_ composites. With these successes, MAX phase densified Si_3_N_4_ ceramics with enhanced strength and toughness will be necessary to meet demands of potential future markets for advanced ceramics. Further efforts are encouraged to be devoted to thermal properties investigations of MAX enabled Si_3_N_4_ composites.

## Figures and Tables

**Table 1:** Refinement parameters of Ti_3_SiC_2_ doped Si_3_N_4_ ceramics.

Si_3_N_4−*x*_ vol.% Ti_3_SiC_2_	Weight Fraction			*R* Factor of Rietveld	Theoretical Density (g·cm^−3^)	Densification
	*β*-Si_3_N_4_	C_0.3_N_0.7_Ti	SiC			
*x* = 1	97.3%	0.9%	1.8%	4.76%	3.21	99.26%
*x* = 3	97.1%	0.9%	2.1%	4.55%	3.21	99.89%
*x* = 5	92.8%	4.1%	3.1%	3.46%	3.25	99.92%
*x* = 10	87.3%	7.6%	5.1%	3.42%	3.30	99.73%
*x* = 15	82.3%	11.7%	6.0%	3.47%	3.35	99.98%
*x* = 20	78.4%	14.6%	7.0%	4.07%	3.39	99.79%

**Table 2:** EDS chemical analysis (at.%) at different positions in Figure 3g.

Location	Si	N	O	Ti	C	Possible Phases
Spot A	52.21	35.43	2.04	1.29	9.03	Si_3_N_4_, SiC
Spot B	5.10	27.18	-	50.13	17.59	C_0.3_N_0.7_Ti, SiC

**Table 3:** Selected results on mechanical properties of Si_3_N_4_ ceramics by pressure-assisted sintering.

Composition	Sintering Conditions	Vickers Hardness (GPa)	Flexural Strength (MPa)	Fracture Toughness (MPa·m^1/2^)	Ref.
*α*-Si_3_N_4_ + 4 wt.% Al_2_O_3_ + 6 wt.% Y_2_O_3_	Hot isostatic pressing at 1700 °C, 20 MPa, 3 h	16.4	730	6.5	[27]
*α*-Si_3_N_4_ + 5 wt.% Al_2_O_3_ + 5 wt.% Y_2_O_3_	Hot press at 1800 °C, 30 MPa, 1.5 h	16.1	-	5.2	[20]
*α*-Si_3_N_4_ + 4 wt.% Al_2_O_3_ + 6 wt.% Y_2_O_3_	Hot press at 1700 °C, 50 MPa, 1.5 h	17.01	-	-	[21]
*α*-Si_3_N_4_ + 30 vol.% *β*-Si_3_N_4_ whiskers + 5 wt.% Al_2_O_3_ + 5 wt.% Y_2_O_3_ + 5 wt.% CeO_2_	Hot press at 1700 °C, 30 MPa, 30 min	19.0	794	8.6	[22]
*α*-Si_3_N_4_+4 wt.% Al_2_O_3_+4 wt.% Y_2_O_3_+15 vol.% SiC whiskers	Hot press at 1800 °C, 30 MPa, 30 min	16	680	6.1	[68]
*α*-Si_3_N_4_ + 5 wt.% Al_2_O_3_ + 4 wt.% Y_2_O_3_ + 3 wt.% TiC	Gas pressure sintering at 1750 °C, 2 MPa	16.4	475	7.6	[11]
*α*-Si_3_N_4_ + 5 vol.% Ti_3_SiC_2_	Hot-pressed at 1700 °C for 90 min	19.78	795	4.88	This work
*α*-Si_3_N_4_ + 20 vol.% Ti_3_SiC_2_	Hot-pressed at 1700 °C for 90 min	20.11	549	6.58	This work

## Supplementary Materials

The following are available online at https://www.mdpi.com/1996-1944/13/6/1428/s1, Figure S1: Quantitative report of Si_3_N_4_-1 vol.% Ti_3_SiC_2_, Figure S2. Quantitative report of Si_3_N_4_-3 vol.% Ti_3_SiC_2_, Figure S3. Quantitative report of Si_3_N_4_-5 vol% Ti_3_SiC_2_, Figure S4. Quantitative report of Si_3_N_4_-10 vol.% Ti_3_SiC_2_, Figure S5. Quantitative report of Si_3_N_4_-15 vol.% Ti_3_SiC_2_, Figure S6. Quantitative report of Si_3_N_4_-20 vol.% Ti_3_SiC_2_.
