# Supplementary material for: C0.3N0.7Ti-SiC Toughed Silicon Nitride Hybrids with Non-Oxide Additives Ti3SiC2
Source: Materials (Basel). 2020 Mar 20;13(6):1428. doi: 10.3390/ma13061428 (PMC7143876; doi:10.3390/ma13061428)
Supplement: Supplementary file 1 [file materials-13-01428-s001.pdf]

Supplementary Materials

Detailed information concerning quantitative report of Si_3N_4, $C_{0.3}N_{0.7}Ti$, and SiC are illustrated as follow:

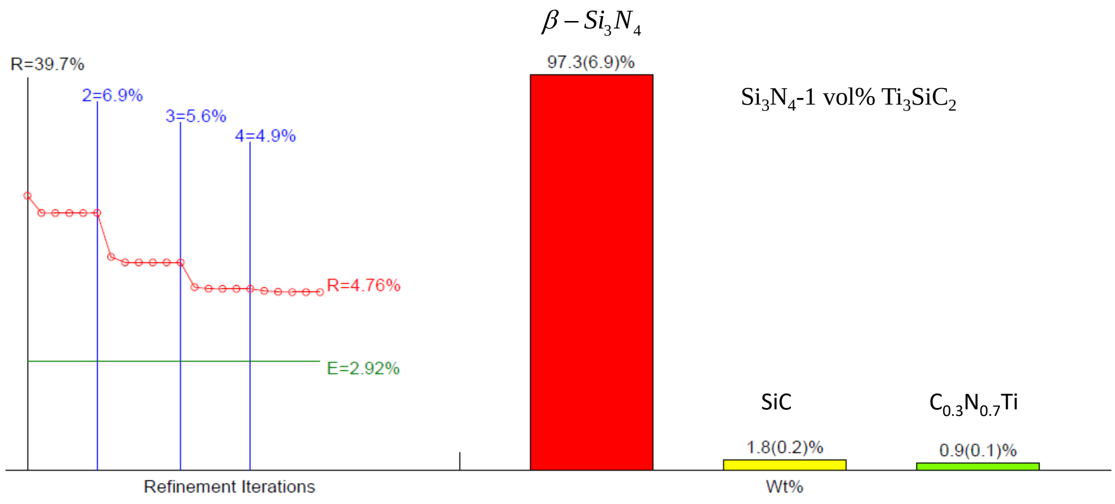

Figure S1. Quantitative report of Si_3N_4-1 vol.% Ti_3SiC_2.

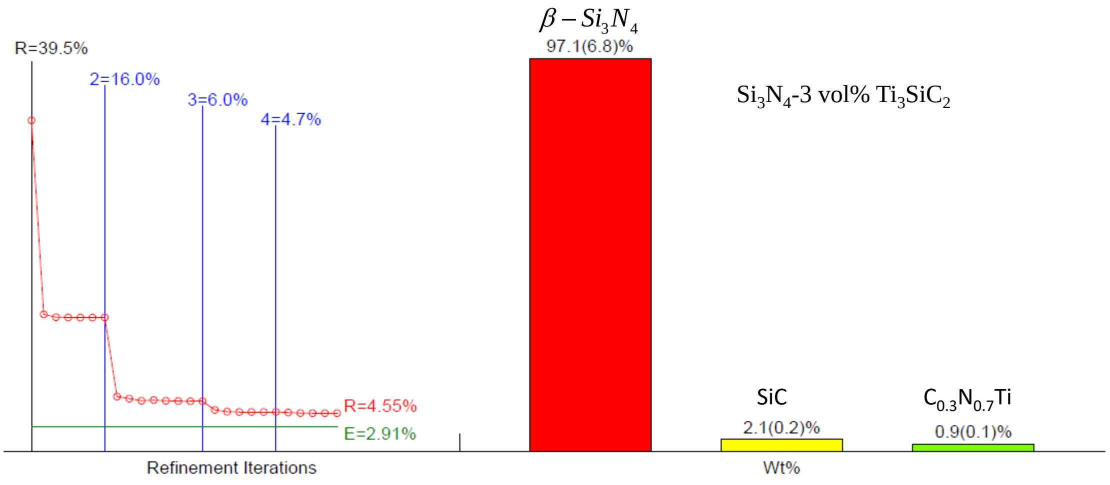

Figure S2. Quantitative report of Si_3N_4-3 vol.% Ti_3SiC_2

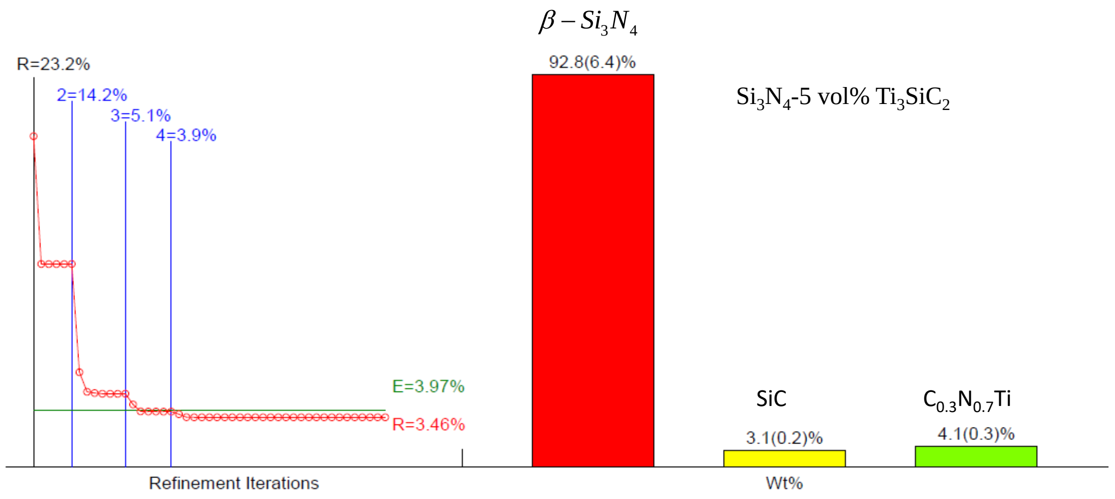

Figure S3. Quantitative report of Si_3N_4-5 vol% Ti_3SiC_2.

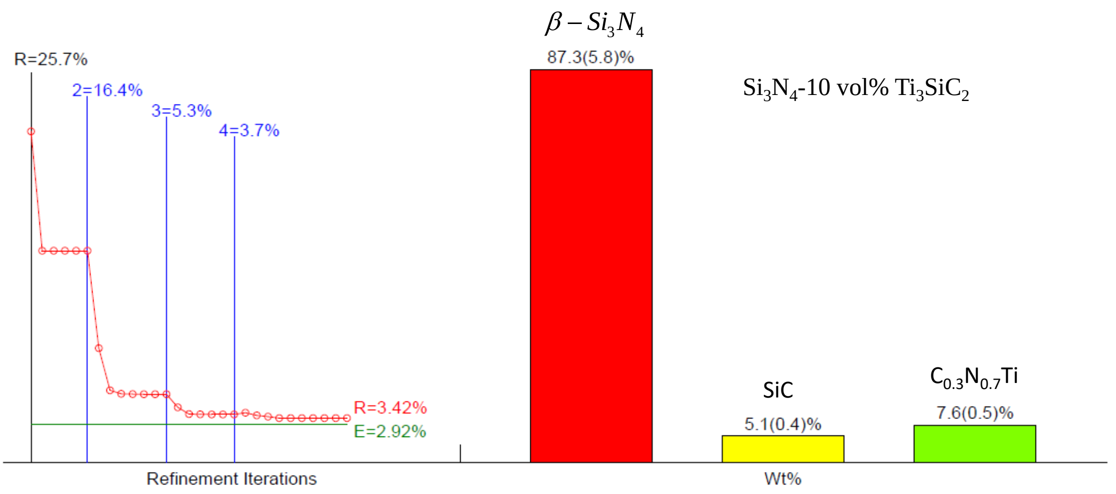

Figure S4. Quantitative report of Si_3N_4-10 vol.% Ti_3SiC_2

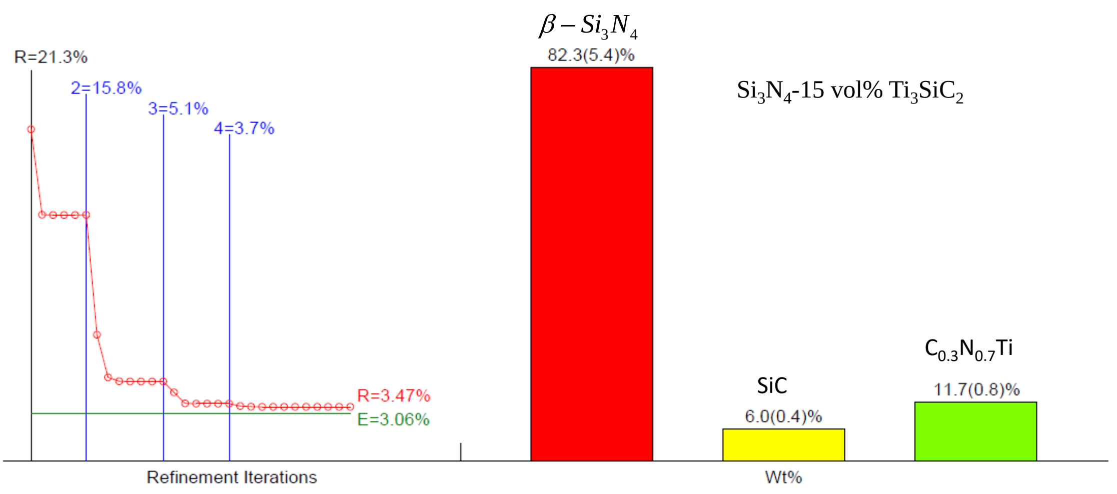

Figure S5. Quantitative report of Si_3N_4-15 vol.% Ti_3SiC_2.

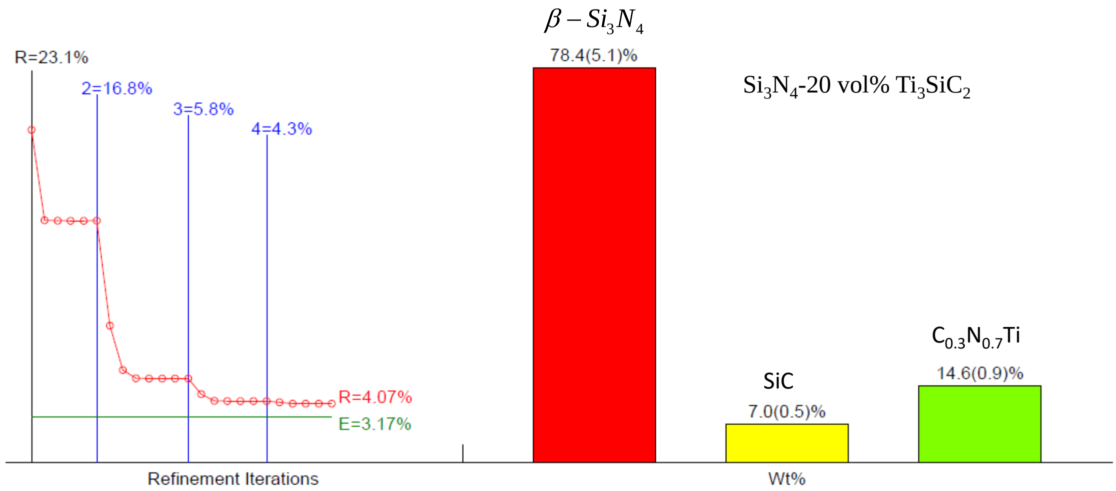

Figure S6. Quantitative report of Si_3N_4-20 vol.% Ti_3SiC_2.
